# Supplementary material for: Orbital topological edge states and phase transitions in one-dimensional acoustic resonator chains
Source: Nat Commun. 2023 Dec 9;14:8162. doi: 10.1038/s41467-023-44042-z (PMC10710482; doi:10.1038/s41467-023-44042-z)
Supplement: Supplementary file 1 — Supplementary Information [file 41467_2023_44042_MOESM1_ESM.pdf]

Supplementary Information for

"Orbital topological edge states and phase transitions in one-dimensional acoustic resonator chains"

Feng Gao[1,*], Xiao Xiang[1,*], Yu-Gui Peng[1,†], Xiang Ni[2,3], Qi-Li Sun[1], Simon Yves[3], Xue-Feng Zhu[1,†], Andrea Alù[3,4,†]

[1]*School of Physics and Innovation Institute, Huazhong University of Science and Technology, Wuhan 430074, China*

[2]*School of Physics and Electronics, Central South University, Changsha, 410083, China*

[3]*Photonics Initiative, Advanced Science Research Center, City University of New York, New York, NY 10031, USA*

[4]*Physics Program, Graduate Center, City University of New York, New York, NY 10016, USA*

[*] These authors contributed equally to this work.

[†]Corresponding authors: ygpeng@hust.edu.cn (Y.-G.P.); xfzhu@hust.edu.cn (X.-F. Z.); aalu@gc.cuny.edu (A.A.)

Contents

Supplementary Note 1. *Amplitude and phase responses in a dimer-unit* 2

Supplementary Note 2. *Analysis of the coupling parameters* 3

Supplementary Note 3. *Hamiltonians, phase transitions and chiral symmetry* 6

Supplementary Note 4. *The hidden duality symmetry* 13

Supplementary Note 5. *Numerical field profiles for different bonding angles* 16

Supplementary Note 6. *Numerical responses for different orbital sources* 18

Supplementary Note 7. *Topological robustness against disorders* 19

Supplementary Note 8. *Numerical results for the counterintuitive edge modes* 22

Supplementary Note 9. *Details for simulations and experiments* 24

Supplementary Note 1. *Amplitude and phase responses in a dimer-unit.*

In the main text, we have shown the experimentally measured pressure amplitude responses of the $\boldsymbol{\sigma}$ coupling and $\boldsymbol{\pi}$ coupling in the coupled dimer sample and plotted the corresponding pressure amplitude spectra in Fig. 1. For comparison, here we present the simulation result in Fig. S1**a**, which is calculated by the pressure acoustic module in COMSOL Multiphysics. Considering the inevitable dispassion loss, we introduce the speed $c' = 1.0\ \mathrm{m/s}$ to the imaginary part of sound speed in the simulations.

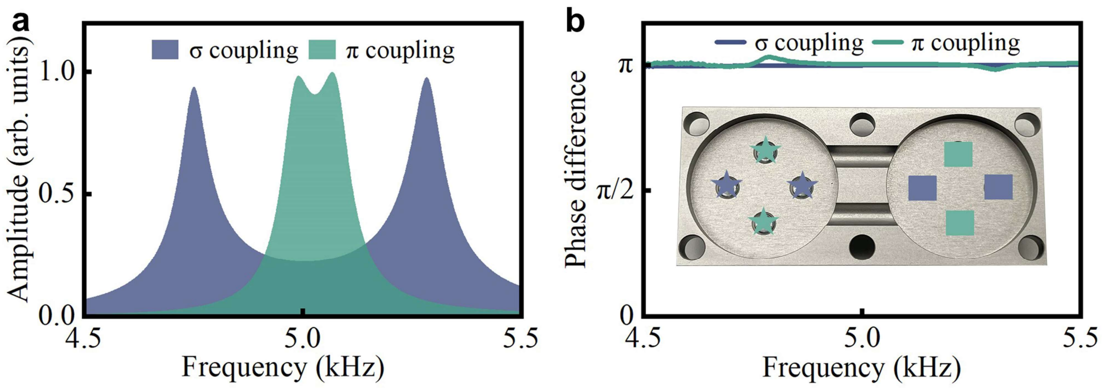

Fig. S1. (a) Simulated amplitude spectra corresponding to $\boldsymbol{\sigma}$ coupling and $\boldsymbol{\pi}$ coupling in a coupled dimer structure. (b) Experimentally measured phase spectra. Inset shows the photograph of the fabricated dimer structure in experiments.

We find that the simulated spectra agree well with the experimentally measured ones. The resonance peak shift is mainly caused by the machining errors as well as the sound leakage. As shown by Fig. 1 in the main text, the acoustic pressure fields of ***p***-orbital eigenmodes in the coupled dimer structure are polarized with an exact π phase difference. We experimentally demonstrated this by using the fabricated dimer structure in Fig. S1**b**, where the measured phase spectra of $\boldsymbol{\sigma}$ coupling and $\boldsymbol{\pi}$ coupling are also displayed. In the experiment, we launched an orbital sound signal with the orientation highlighted by the dark blue (green) stars and measured the phase responses in the holes marked by dark blue (green) squares. The phase derived inside one of the holes is set as the reference. In Fig. S1**b**, the measured spectrum for $\boldsymbol{\sigma}$ ($\boldsymbol{\pi}$) coupling is clearly featured with a polarization of unambiguous π phase difference.

Supplementary Note 2. *Analysis of the coupling parameters.*

Firstly, we explore the variation of eigenfrequencies and coupling strengths for dimer-units coupled with tubes of different diameters. We perform simulations to analyze the influences for the **σ**-coupled modes and **π**-coupled modes when the diameters of coupling tubes vary in the range [1 mm, 8 mm]. The obtained results are shown in Fig. S2. As shown in Fig. S2**a**, the eigen-frequencies of **σ** bonding (**σ** anti-bonding) and **π** bonding (**π** anti-bonding) both decrease (increase) as the diameter of the tube d varies from 1 mm to 8 mm. The eigenfrequencies of **σ** bonding (**π** bonding) are nearly symmetrical to the single-resonator eigenfrequency around 5028.5Hz.

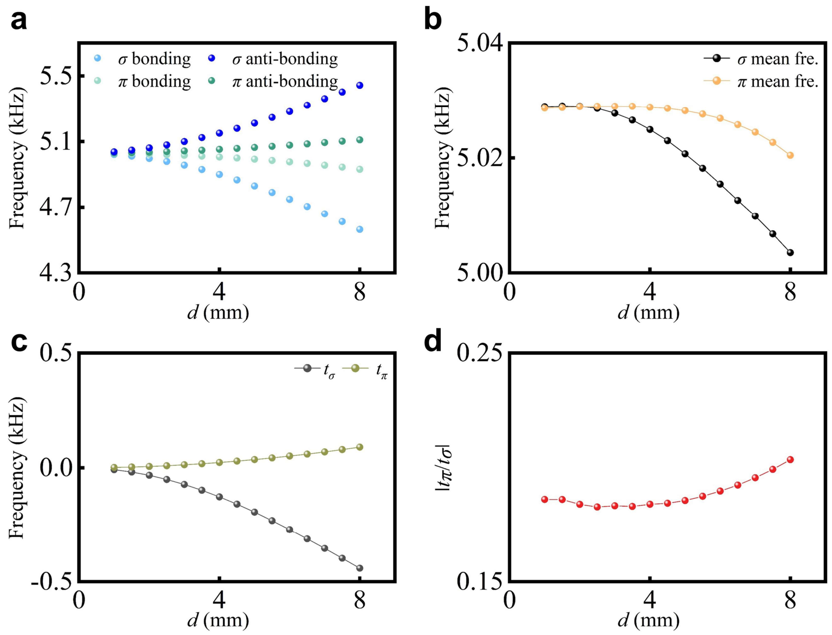

Fig. S2. (a) Simulated eigen-frequencies for the dimer-units with the diameters of coupling tubes varying from 1 mm to 8 mm. The modes for **σ** bonding, **π** bonding, **π** anti-bonding, and **σ** anti-bonding are denoted by light blue, light green, green and blue spheres, respectively. (b) The mean frequencies of the **σ**-coupled modes and **π**-coupled modes. (c) The coupling coefficients for t_σ and t_π. (d) The contrast ratio.

The relations for the mean frequencies of the **σ**-coupled modes and **π**-coupled modes with the varying diameter d of coupling tubes are displayed in Fig. S2**b**. The mean frequency of the **σ**-coupled modes (black dotted line) and the **π**-coupled modes

(orange dotted line) are almost identical as $d < 3$ mm. The mean frequency difference between the $\boldsymbol{\sigma}$-coupled modes and $\boldsymbol{\pi}$-coupled modes rapidly increases as $d > 3$ mm. In our simulations and experiments, we choose the diameter of the coupling tubes as $d = 6$ mm. The coupling strengths t_σ and t_π are proportional to half of the difference between the two related eigenfrequencies for the coupled dimer unit. The extracted effective coupling strengths have opposite signs: the values for t_σ and t_π are negative and positive, respectively[1]. The coupling coefficients t_σ and t_π vary with the diameter d, as exhibited in Fig. S2**c**. Clearly, the coupling strengths of t_σ and t_π increase as d varies from 1 mm to 8 mm, and t_σ increases faster than t_π. The absolute contrast ratio between t_π and t_σ is also plotted in Fig. S2**d**.

Next, we fix the diameter of coupling tubes to be $d = 6$ mm and investigate the influence of the interval between cavities or coupling tubes on the frequency spectra of the coupled resonators. In the first scenario, we vary the interval (w_2) between the dimer resonators (the length l of the paired coupling tubes varies accordingly), and perform simulations as l varied from 0 mm to 26 mm while fix the coupler interval as $w_1 =$ 16mm. Here we define $l = w_2 - D$ and l = 0mm when the interval between two cavities is zero. The numerically calculated eigen-spectra for $\boldsymbol{\sigma}$ bonding, $\boldsymbol{\pi}$ bonding, $\boldsymbol{\pi}$ anti-bonding, and $\boldsymbol{\sigma}$ anti-bonding modes are shown in Fig. S3**a**. Clearly, the eigen-frequencies decrease as l varies from 0mm to 26mm. The coupling coefficients are furtherly depicted in Fig. S3**b.** t_σ gradually decreases as $l < 11$ mm, and then increases as $l > 11$ mm, implying a critical point at $l = 11$ mm. For t_π, the critical point is around $l = 8.5$mm. The absolute contrast ratio of t_π / t_σ at each interval l is exhibited in Fig. S3**c**. For $l < 15$ mm, the contrast ratio varies very slowly but rapidly increases with l when $l > 15$ mm.

In the second scenario, we keep the interval between cavities $l = 13$mm and vary the coupler interval parameter w_1. For convenience, we set $w = w_1 / 2$ and investigate the variation of eigenfrequencies and coupling strengths for $w \in [3\text{ mm}, 13\text{ mm}]$. The numerically simulated eigen-spectra, coupling coefficients, and $|t_\pi / t_\sigma|$ are displayed in Figs. S3**d-3f**, respectively. The mode frequencies for $\boldsymbol{\sigma}$ bonding, $\boldsymbol{\pi}$ bonding, and $\boldsymbol{\sigma}$ anti-

bonding tend to decrease, while the mode frequency for $\boldsymbol{\pi}$ anti-bonding appears to increase, as shown in Fig. S3**d**. The absolute value of coupling coefficient t_σ decreases as w varies in the range [3 mm,13 mm], while the value t_π keeps increasing, as displayed in Fig. S3**e**. Based on the above discussed, the coupling strengths for t_σ and t_π can be tailored by virtue of the variation of d, l and w, and the synergistic effect for the variation of l and w as well as d can be considered to obtain the coupling coefficient in demand.

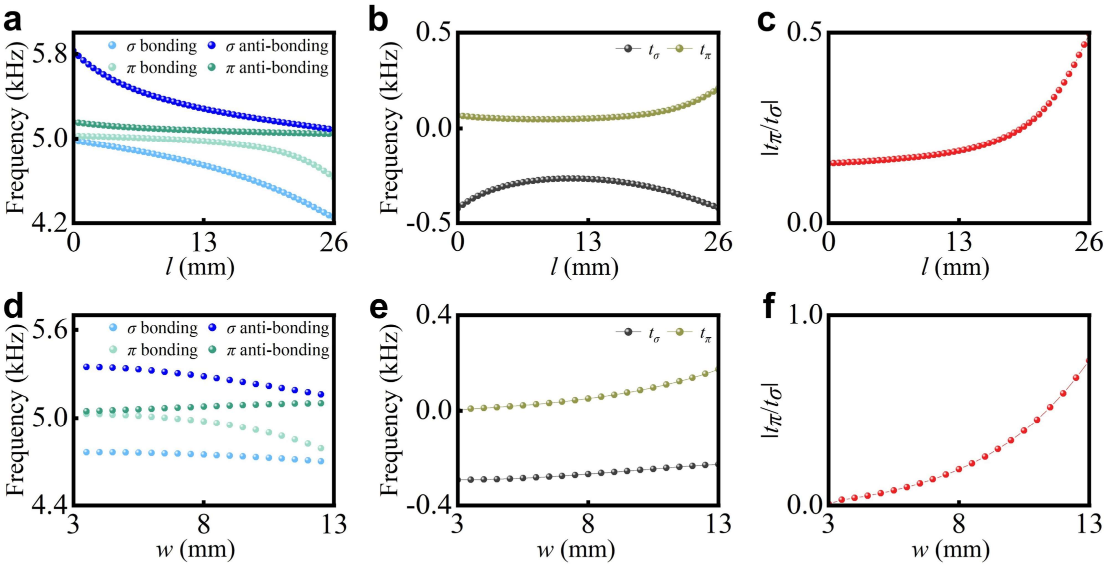

Fig. S3. (a) Simulated eigen-frequencies for the dimer units with the interval (l) between two resonators varying in the range [0 mm, 26 mm]. The interval is 13 mm for the sample in the main text. The modes for $\boldsymbol{\sigma}$ bonding, $\boldsymbol{\pi}$ bonding, $\boldsymbol{\pi}$ anti-bonding, and $\boldsymbol{\sigma}$ anti-bonding are denoted by light blue, light green, green and blue spheres, respectively. (b) The coupling coefficients for t_σ and t_π. (c) The absolute contrast ratio between $|t_\pi / t_\sigma|$ with l. (d) Simulated eigen-spectra for the dimer units with varying w in the range [3 mm, 13 mm]. (e) The coupling coefficients of t_σ and t_π. (f) The absolute contrast ratio $|t_\pi / t_\sigma|$ with different w.

Supplementary Note 3. *Hamiltonians, phase transitions and chiral symmetry.*

S3.1 *Orbital Hamiltonians in infinite and finite lattices*

We firstly present a detailed derivation of the orbital Hamiltonian. Under the scenario of tight-binding approximation, the annihilate operator corresponding to the ***p*** orbital can be defined on the p_x and p_y basis $\vec{p}=\left(p_x, p_y\right)^T$. For the longitudinal hopping, the ***p*** orbital should be projected along the hopping orientations of the lattice, *i.e.*, the $e_{1,2}$ in Fig. S4. Two types of projected operators read as

$$a_n b_n = \vec{e}_1 \cdot \vec{p} = -p_y \,, \tag{1}$$

$$a_{n+1} b_n = \vec{e}_2 \cdot \vec{p} = \sin(\theta) p_x + \cos(\theta) p_y \,. \tag{2}$$

while for the transverse hopping, the ***p*** orbital should be projected along orientations perpendicular to $e_{1,2}$, and the corresponding projected operators read as

$$a'_n b'_n = \vec{d}_1 \cdot \vec{p} = p_x \,, \tag{3}$$

$$a'_{n+1} b'_n = \vec{d}_2 \cdot \vec{p} = -\cos(\theta) p_x + \sin(\theta) p_y \,. \tag{4}$$

The real-space Hamiltonian of the orbital-dependent lattice reads as

$$H = \sum_n \left[t_\sigma \left(a^\dagger_{n,\sigma} b_{n,\sigma} + a'_{n+1,\sigma} b'^\dagger_{n,\sigma} \right) + t_\pi \left(a^\dagger_{n,\pi} b_{n,\pi} + a'_{n+1,\pi} b'^\dagger_{n,\pi} \right) \right] + h.c. \,, \tag{5}$$

The basis transformation:

$$\begin{pmatrix} a(b)_{n,\sigma} \\ a(b)_{n,\pi} \end{pmatrix} = \hat{R} \begin{pmatrix} a(b)_{n,x} \\ a(b)_{n,y} \end{pmatrix}, \tag{6}$$

$$\begin{pmatrix} a'(b')_{n,\sigma} \\ a'(b')_{n,\pi} \end{pmatrix} = \hat{R}' \begin{pmatrix} a(b)_{n,x} \\ a(b)_{n,y} \end{pmatrix}, \tag{7}$$

here

$$\hat{R} = \begin{pmatrix} 0 & -1 \\ 1 & 0 \end{pmatrix}, \quad \hat{R}' = \begin{pmatrix} \sin(\theta) & \cos(\theta) \\ -\cos(\theta) & \sin(\theta) \end{pmatrix}$$

In Fourier basis $\Phi(\Phi') = [a(a')_{\mathbf{k},\sigma}, a(a')_{\mathbf{k},\pi}, b(b')_{\mathbf{k},\sigma}, b(b')_{\mathbf{k},\pi}]^T$, the Hamiltonian reads

$$H = \sum_{\mathbf{k}} (\Phi^\dagger H_1(\mathbf{k}) \Phi + \Phi'^\dagger H_2(\mathbf{k}) \Phi') \,, \tag{8}$$

where

$$H_1(\mathbf{k}) = \begin{pmatrix} & & t_\sigma & \\ & & & t_\pi \\ t_\sigma^* & & & \\ & t_\pi^* & & \end{pmatrix}, H_2(\mathbf{k}) = H_1(\mathbf{k}) e^{i\mathbf{k}}.$$

After the unitary transformation with the respective basis $\psi = [a_{\mathbf{k},x},\ a_{\mathbf{k},y},\ b_{\mathbf{k},x},\ b_{\mathbf{k},y}]^T$, the kernel of new Hamiltonian becomes

$$H(\mathbf{k}) = UH_1(\mathbf{k})U^{-1} + U'H_2(\mathbf{k})U'^{-1} \tag{9}$$

where

$$U = I_{2\times2} \otimes \hat{R}, U' = I_{2\times2} \otimes \hat{R}'.$$

To be specific, the matrix $H(\mathbf{k})$ is

$$H(\mathbf{k}) = \begin{pmatrix} 0 & 0 & D_1 & D_2 \\ 0 & 0 & D_2 & D_4 \\ D_1^* & D_2^* & 0 & 0 \\ D_2^* & D_4^* & 0 & 0 \end{pmatrix},$$

$$D_1 = t_\pi + [t_\sigma \sin^2(\theta) + t_\pi \cos^2(\theta)] e^{i\mathbf{k}},$$

$$D_2 = (t_\pi - t_\sigma) \sin(\theta) \cos(\theta) e^{i\mathbf{k}},$$

$$D_4 = t_\sigma + [t_\pi \sin^2(\theta) + t_\sigma \cos^2(\theta)] e^{i\mathbf{k}}. \tag{10}$$

The $a(b)_{n,\sigma}$ and $a(b)_{n,\pi}$ correspond to the σ (π) types of projection operators related with two sites a, b in the dimer unit, and t_σ (t_π) denote the longitudinal (transverse) hopping strengths, and $I_{2\times2}$ denotes a 2×2 identity matrix.

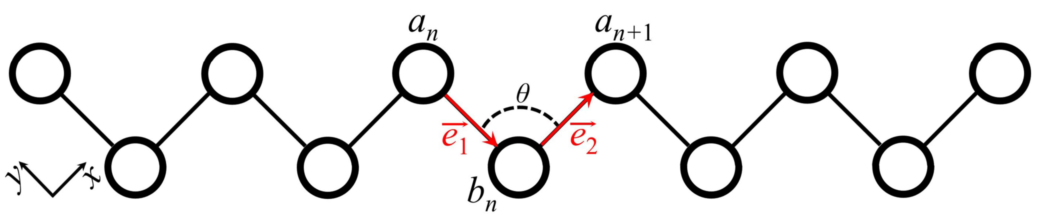

Fig. S4. Illustration of the 1D orbital SSH model for the infinite lattice, and $\vec{e}_1$, $\vec{e}_2$ denote two unit-vectors along the hopping directions.

In the main text, we utilized the Hamiltonian for finite chain to calculate the energy spectra and edge modes in Fig 2**c** and Fig 2**d**. Generally, in a concrete and practical form, the Hamiltonian for a finite chain is expressed as follows

$$H=\begin{pmatrix} 0 & D_{f} & 0 & 0 & 0 \\ & 0 & D_{f'} & 0 & 0 \\ & & \ddots & \ddots & 0 \\ & & & 0 & D_{f'} \\ & & & & 0 \end{pmatrix},\quad H=H+h.c.,$$

$$D_{f}=\begin{pmatrix} D_{1'} & D_{2'} \\ D_{3'} & D_{4'} \end{pmatrix},\quad D_{f'}=\begin{pmatrix} D_{1''} & D_{2''} \\ D_{3''} & D_{4''} \end{pmatrix},$$

$$D_{1'}=t_{\pi},\quad D_{1''}=t_{\sigma}\sin^{2}(\theta)+t_{\pi}\cos^{2}(\theta),$$

$$D_{2'}=D_{3'}=0,\quad D_{2''}=D_{3''}=(t_{\pi}-t_{\sigma})\sin(\theta)\cos(\theta),$$

$$D_{4'}=t_{\sigma},\quad D_{4''}=t_{\pi}\sin^{2}(\theta)+t_{\sigma}\cos^{2}(\theta). \tag{11}$$

For a chain constituted by 11 cavity resonators in our work, Eq. (S11) takes the form of a 22×22 matrix. By calculating the eigenvalues and eigen-vectors of this Hamiltonian, we can obtain the eigen-spectra and identify the topological phase transitions as shown in Fig 2c.

S3.2 *Phase transitions and topological invariants*

The topological phase transition condition directly predicts the emergence of topological edge states. The detailed derivation is presented as follows. Underpinned by the orbital Hamitonian Eq. (S10), we can resolve the eigenvalues, for which the general forms are obtained as

$$E_1=-\frac{\sqrt{2}}{2}\left(\sqrt{D_1D_1^*+2D_2D_2^*+D_4D_4^*+\sqrt{D_1^2D_1^{*2}+N_1+N_2)}}\right), \tag{12}$$

$$E_2=-\frac{\sqrt{2}}{2}\left(\sqrt{D_1D_1^*+2D_2D_2^*+D_4D_4^*-\sqrt{D_1^2D_1^{*2}+N_1+N_2)}}\right), \tag{13}$$

$$E_3=\frac{\sqrt{2}}{2}\left(\sqrt{D_1D_1^*+2D_2D_2^*+D_4D_4^*-\sqrt{D_1^2D_1^{*2}+N_1+N_2)}}\right), \tag{14}$$

$$E_4 = \frac{\sqrt{2}}{2}\left(\sqrt{D_1 D_1^* + 2D_2 D_2^* + D_4 D_4^* + \sqrt{D_1^2 D_1^{*2} + N_1 + N_2)}}\right), \tag{15}$$

with $N_1 = D_1^*(4D_1D_2D_2^* + 4D_2^{\ 2}D_4^* - 2D_1D_4D_4^*)$, $N_2 = D_4(4D_1D_2^{*2} + 4D_2D_2^*D_4^* + D_4D_4^{*2})$.

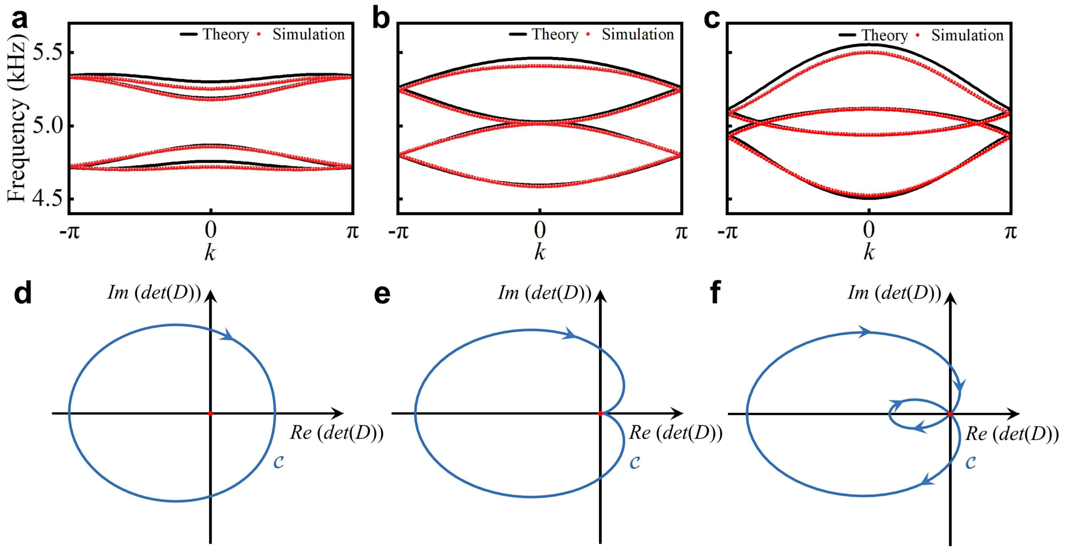

Fig. S5. Calculated and simulated energy spectra marked by black lines and red spheres for different bonding angles at (a) 100°, (b) 132.9°, and (c) 165°. Schematic sketches for a contour C in the complex plane that $\det D$ follows as *k* changes from $-\pi$ to π. (d) Scenario for $|\theta - \pi/2| < \arcsin|(\gamma+1)/(\gamma-1)|$. (e) Scenario for $|\theta - \pi/2| = \arcsin|(\gamma+1)/(\gamma-1)$. (f) Scenario for $|\theta - \pi/2| > \arcsin|(\gamma+1)/(\gamma-1)|$.

We can find that the spectra of Hamiltonian are symmetric with respect to zero. As a result, in the scenario where $\mathbf{k} = 0$, we can obtain the transition points at E_2=0 or E_3=0, and further derive $|\cos\theta| = |(\gamma+1)/(\gamma-1)|$. It clearly shows that the topological transition condition is intimately correlated with the ratio of coupling strengths, *viz.*, $\gamma = t_\sigma / t_\pi$. After some mathematical manipulations, we can derive the phase transition condition of $|\theta - \pi/2| = \arcsin|(\gamma+1)/(\gamma-1)|$. As shown in Fig. S5**a**, the spectrum possesses a band gap, as the bonding angle $|\theta - \pi/2| < \arcsin|(\gamma+1)/(\gamma-1)|$. When the critical condition is satisfied at $|\theta - \pi/2| = \arcsin|(\gamma+1)/(\gamma-1)|$, the gap is just

closed, as shown in Fig. S5**b**. When $|\theta-\pi/2|>\arcsin|(\gamma+1)/(\gamma-1)|$, bulk bands are overlapped without any band gaps, as shown in Fig. S5**c**. All the simulated spectra agree well with the theoretical results on the whole.

The topological properties of the orbital Hamiltonian are characterized by winding number defined by[2]

$$\mathcal{W}=\frac{i}{2\pi}\int_{-\pi}^{\pi}dk\frac{d\ln\det D(k)}{dk}=-\frac{1}{2\pi}\int_{C}d\arg\det D(k) \qquad (16)$$

where $\mathcal{C}$ represents a contour swept by $D(k)$ as k varies across the Brillouin zone. For $|\theta-\pi/2|<\arcsin|(\gamma+1)/(\gamma-1)|$ where the system is gapped as shown in Fig. S5**a**, the contour of $\det D$ forms one turn around zero point, as shown in Fig. S5**d**, and thus we obtain the topological invariant $\mathcal{W}=1$. For the critical condition at $|\theta-\pi/2|=\arcsin|(\gamma+1)/(\gamma-1)|$, the contour for $\det D$ is displayed in Fig. S5**e**, and the winding number is ill-defined. When the band is gapless as shown in Fig. S5**c**, $\det D$ actually passes twice through zero point defined by $\det D=0$, which is shown in Fig. S5**f**. To formally define the winding number, the contour in Fig. S5**f** can be shifted by an infinitesimal amount to the left or to the right, for which we obtain the topological invariant $\mathcal{W}=0$.

For the topological invariants of the orbital SSH model, we can also illustrate the winding numbers from the conventional SSH models[3]. A standard SSH Hamiltonian reads

$$H_{SSH}=\sum_{n}(ta_n b_n^{\dagger}+t'a_n^{\dagger}b_n)+h.c. \qquad (17)$$

where $t(t')$ represents the intracell (intercell) hopping amplitude, and $a_n^{\dagger}(b_n^{\dagger})$ is the creation operator on the site $a_n(b_n)$ in the nth unit cell. H_{SSH} displays two topologically distinct phases as two different dimerizations $t>t'$ and $t<t'$ are considered. The different topology of the two phases is unveiled by considering the winding $\mathcal{W}$ of phase $\phi(k)$ across the Brillouin zone:

$$\mathcal{W} = \frac{1}{2\pi}\int_{BZ}\frac{\partial\phi(k)}{\partial k}dk \tag{18}$$

which corresponds to the Zak phase divided by π. For $t > t'$, the intracell coupling is stronger than intracell coupling, as shown in Fig. S6**a**, corresponding to the trivial case with $\mathcal{W} = 0$. When we cut the lattice into a finite array and keep the stronger couplings on the end, no edge modes will appear. In contrast, for $t < t'$ depicted in Fig. S6**b**, we anticipate nontrivial edge modes on the ends with weaker couplings and the winding number $\mathcal{W} = 1$.

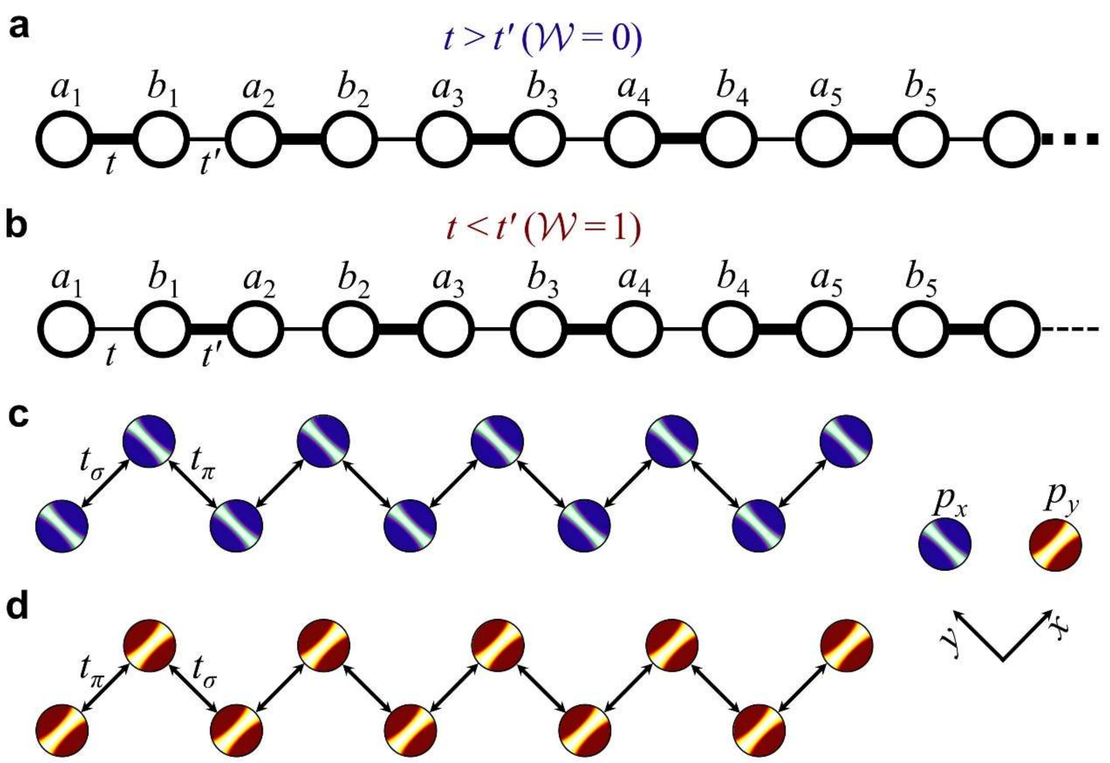

Fig. S6. (a) and (b) Schematic sketches for the two different dimerizations in the conventional SSH models. (c) and (d) Schematic sketches corresponding to the p_x and the p_y subspaces of the orbital SSH models. Each subspace displays similar coupling arrangements as the conventional SSH models.

For the zigzag chain in Fig 2**a** of the main text, the couplings are subjected to alternating strengths along the chain for p_x or p_y orbital, each of which corresponds to one copy of conventional SSH model. In the zigzag arrays with bonding angle $\theta = 90°$

shown in Figs. S6**c** and 6**d**, p_x and p_y orbitals are separately oriented along the diagonal and antidiagonal axes, and the hopping amplitude between consecutive resonators strongly relies on the orientation of the axis connecting the resonators. The hopping strengths longitudinal and transverse to the bond between the cavities are denoted as t_σ and t_π, respectively, and the magnitude of t_σ is larger than that of t_π in our design. Then, as the subspace of p_x mode is excited (Fig. S6**c**), effective dimerized coupling pattern emerges along the chain. In this subspace, effective stronger coupling t_σ ($t_\sigma > t_\pi$) appears on the end, and no edge states are expected to emerge (Fig. S6**c**), which corresponds to the trivial topological phase ($\mathcal{W} = 0$). For the p_y subspace depicted in Fig. S6**d**, it terminates with weaker coupling t_π, and topological edge states are anticipated on the boundaries, corresponding to the nontrivial topological phase ($\mathcal{W} = 1$).

S3.3 *Chiral symmetry in the orbital SSH models*

For the orbital SSH models with bonding angle θ = 90° shown in Figs. S6**c** and S6**d**, they also share chiral symmetry as the conventional ones[3,4]. There exists a unitary transformation U_c that anticommutes with the Hamiltonian: $\{H, U_c\} = 0$. For the p_x/ p_y subspace SSH lattice with bonding angle θ = 90° as discussed above, the Hamiltonian of the system (Eq. (S17)) can be expressed in the momentum space

$$H(k) = \vec{d}(k) \cdot \vec{\sigma}, \tag{19}$$

where $\sigma_{x,y,z}$ are the Pauli matrices, and

$$d_x(k) = t + t'cos(ka), d_y(k) = t'sin(ka), d_z(k) = 0 \tag{20}$$

with a being the unit-cell constant[5]. The chiral operator is defined by the Pauli matrix σ_z, namely $\{H, \sigma_z\} = 0$. Thus, the chiral symmetry of orbital SSH Hamiltonian is preserved. On the other hand, for the general orbital Hamiltonians with arbitrary bonding angles as described by Eq. (2) in the main text, it also obeys the generalized chiral symmetry as $\{H, \sigma_z \otimes \sigma_0\} = 0$.

Supplementary Note 4. The *hidden duality symmetry*.

In general, symmetries including the spatial symmetry and internal symmetry are transformation commuting with the Hamiltonian, which makes system invariant after the transformation. Duality plays the same role as the symmetry, which however also enables the parameters vary in a certain pattern, thus mapping a system to another one with the same eigen-energy spectrum. In fact, duality is the hidden symmetry that maps different physical systems between one another in a counterintuitive way [6,7]. In math, it can be stated that the family of Hamiltonian $H(p)$ is endowed with duality by satisfying $UH(t(p))U^{-1} = H(p)$, where $H(p)$ is the Hamiltonian of the system with a parameter p, U is a unitary operator, and t is a map from the parameter space P to itself. Here P represents the parameter space where the parameter p locates. The duality is determined by the cooperation of U and t. The fixed points of the map t are termed self-dual points, where $H(p) \equiv H(t(p))$ and the duality reduces to a symmetry.

In a spatially periodic system, harnessing the Bloch Hamiltonian is an efficient and convenient way to characterize the duality, which embodies in a concrete form of

$$U(\Theta k)H(t(p),\Theta k)U^{-1}(\Theta k) = H(p,k), \tag{21}$$

where k is the quasi-momentum in the Brillouin zone, Θ represents an orthogonal matrix, and the duality generally changes k to Θk. Here it should be emphasized that k does not play the same role as the external parameter p. To be specific, the entire family $k \to H(p,k)$ with different k just characterizes only one physical system, whereas the family with different p corresponds to different physical systems.

In a 1D system with two degrees of freedom (DoFs) in each unit cell, the duality transformation takes the form of $U = \begin{pmatrix} i\sigma_y & 0 \\ 0 & i\sigma_y T(a_1) \end{pmatrix} \Xi$, where σ_y represents the Pauli matrix, $T(\beta)$ is the translation operator by a Bravais-lattice vector β, a_1 is a primitive vector of the Bravais lattice, and $\Xi = \sum_{\gamma} |-\beta\rangle\langle\beta|$. This corresponds to Eq.

(S21) in the momentum space with $U(k)=\begin{pmatrix} i\sigma_y & 0 \\ 0 & i\sigma_y e^{-ik\cdot a_1} \end{pmatrix}$ and $g(k)=-k$, where g represents an arbitrary space group operation. To guarantee the spectra of self-dual Hamiltonians to be two-fold degenerated in the whole Brillouin zone, we require some additional conditions. To be specific, the dualities are related with a one-dimensional parameter space $P=\mathbb{R}$ and $t(p)=-p$. Therefore, we require an anti-unitary operator $A=U\mathcal{K}$ that satisfies $A^2=-1$, which can be obtained by the combination of the duality operation U with the complex conjugation $\mathcal{K}$. Since the energy spectra of the Hamiltonians must be two-fold degenerate over the Brillouin zone at the self-dual points, duality is absent in one-DoF systems. For two-DoFs orbital-dependent systems, the situation is very different, as shown by the band structures for different bonding angles in Fig. 2**b** of the main text.

In addition, we have also explored other physical consequences endowed by the duality symmetry and performed the corresponding simulations. We have numerically calculated the eigenfrequency spectra and edge modes of the zigzag chains in two cases. One is a chain with the same bonding angle θ (Case1), and the other is a chain with θ on the left part and the corresponding dual one $2\theta_c-\theta$ on the right part (Case2). The results are shown in Fig. S7. As displayed in Figs. S7**a**-7**c**, all the eigenfrequencies for Case 1 and Case 2 are almost identical as θ are 70°, 75° and 80°, respectively. The duality symmetry protects the topological properties when the orbital system has the disorders obeying duality symmetry. The eigenfrequencies of edge modes clearly remain unchanged as we switch Case1 to Case2. In fact, any hybrid bonding angles obeying the duality symmetry do not change the eigenfrequency spectra. Furtherly, we have presented the simulated pressure fields of the orbital edge modes for the three hybrid lattices in Figs. S7**d**-7**f**, where the pressure fields are highly localized and polarized with p orbitals on the leftmost edge site with θ and the rightmost edge site with $2\theta_c-\theta$, showing a good agreement with the unitary lattice demonstrated in the main text.

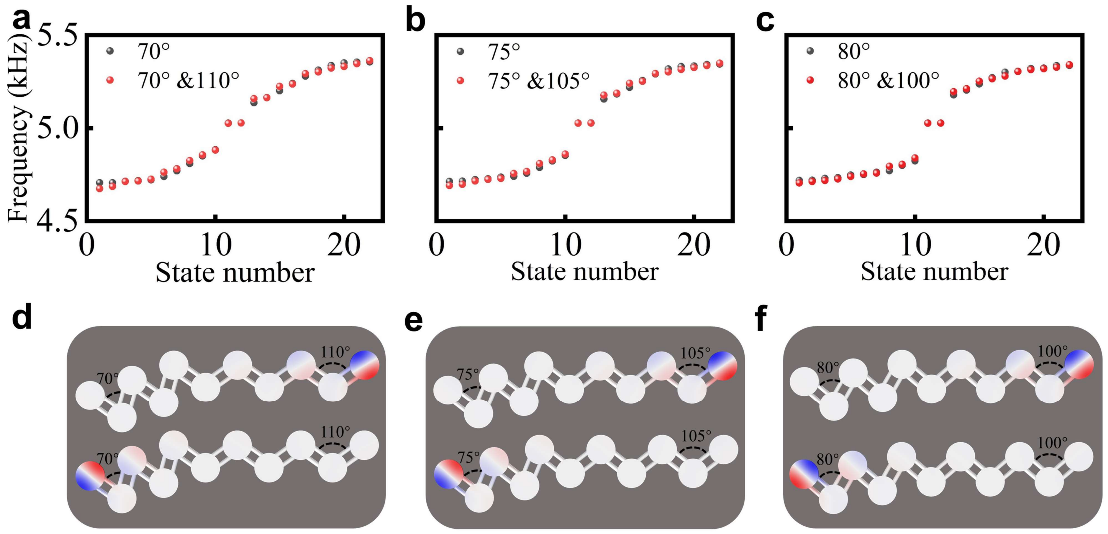

Fig. S7. (a)-(c) The eigenfrequency spectra for the unitary and hybrid chains. (d)-(f) Corresponding topological edge modes, robust against the disorders obeying duality symmetry.

Supplementary Note 5. *Numerical field profiles for different bonding angles*.

Based on Eq. (S11), we have captured the spectrum between the eigen-frequencies and the bonding angle θ for an orbital SSH lattice containing sixty sites, as shown in Fig. 2**c** of the main text. The highlighted solid line in the band gap indicates the emergence of nontrivial edge states, where θ satisfies $|\theta-\pi/2|<\arcsin|(\gamma+1)/(\gamma-1)|$, with $\gamma=t_\sigma/t_\pi$. To show the nontrivial orbital edge states, we display the simulation results for three systems with different bonding angles in Fig. S8. As shown in Figs. S8**a**-8**c**, the pressure fields are highly localized and polarized with ***p*** orbitals in the leftmost edge site and the rightmost edge site, where the bonding angles of the zigzag lattices are 70°, 90°, 110°, respectively, demonstrating the presence of nontrivial orbital edge states and elucidating the phase difference of π for ***p*** orbital modes.

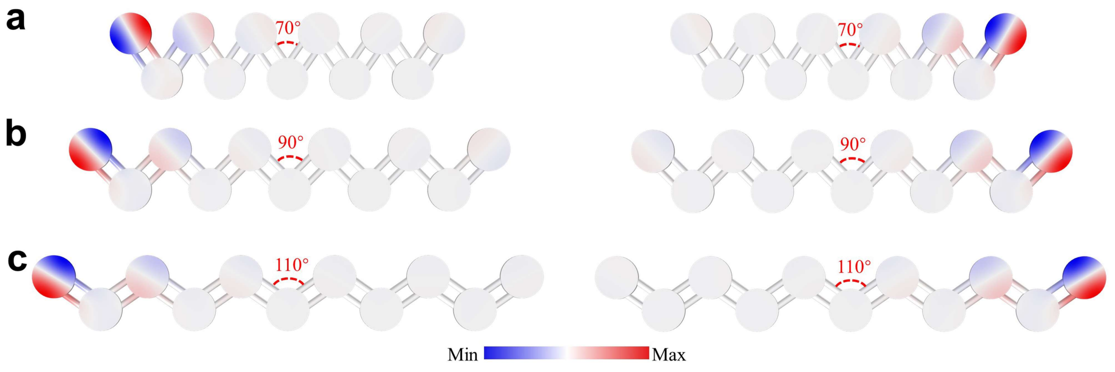

Fig. S8. Acoustic pressure fields for the left-side and right-side nontrivial orbital edge states for the zigzag lattices with different bonding angles of (a) 70°, (b) 90°, and (c) 110°.

The investigated zigzag chain model with bonding angle θ is depicted in the top subfigure of Fig. S9**a**. The smallest accessible θ is 45° in our sample, which is in the trivial regime, and the corresponding eigen-field is shown in the middle subfigure of Fig. S9**a**. In the bottom subfigure of Fig. S9**a**, we display the eigen-field for another trivial case of θ = 180°. To avoid any possible confusion, we furtherly plot the energy spectra for the cases with achievable bonding angles in the realistic zigzag array, and

the ones with unachievable bonding angles are denoted by a grey rectangle as shown in Fig. S9**b**. Due to the hidden duality symmetry, the physics of these unachievable cases are identical to the ones with large bonding angles between 135° and 180°.

On the other hand, we can alternatively design another quasi-two-dimensional array as shown Fig. S9**c**, where arbitrary in-plane bonding angles can be obtained by projection. The unachievable configuration with bonding angle $\theta = 30°$ is depicted as an example in COMSOL. The projective structure in x-y plane is illustrated in Fig. S9**d**. The corresponding eigen-fields at the central frequency are also presented, which explicitly display the trivial features with no edge modes.

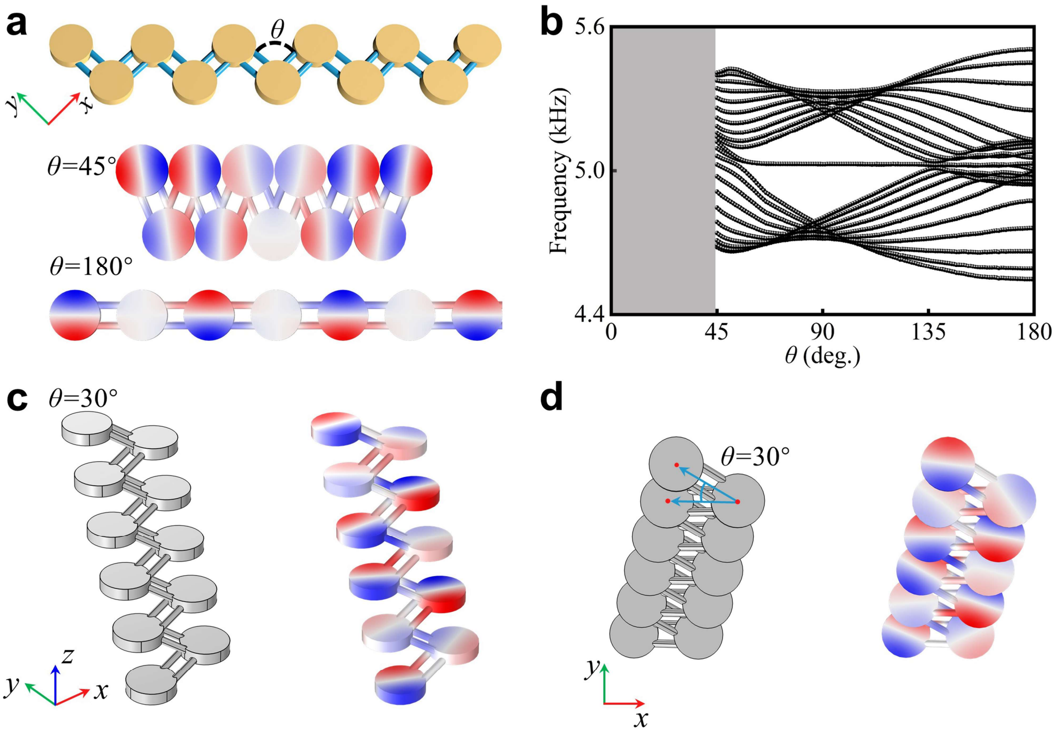

Fig. S9. (a) Schematic of the orbital SSH model and the trivial numerical pressure eigen-fields. Noting that $\theta = 45°$ is the smallest practical angle in this design. (b) The numerical energy spectra. The grey region denotes the unreachable bonding angles. (c) and (d) Alternative quasi-two-dimensional orbital SSH model and eigen-fields with arbitrary bonding angles.

Supplementary Note 6. *Numerical responses for different orbital sources.*

In Fig. 3**b** of the main text, we show the measured amplitude spectra in the edge resonators with the 90° bonding angle, when the orbital sound signals are generated via the L_p_x, L_p_y, R_p_x, and R_p_y excitations, respectively. For comparison, we show the simulated counterparts in Figs. S10**a** and S10 **b**. When sound signals are generated via L_p_x and R_p_y excitations, the acoustic amplitude spectra are featured with prominent peaks in the band gap, corresponding to the frequencies at which nontrivial orbital edge states occur. On the contrary, when the sound signals are generated via L_p_y and R_p_x excitations, the spectra have no peaks in the band gap. All these simulated results match well with the experimental ones. From the above discussions, it can be concluded that the topological orbital edge states can be easily controlled by the excitation orientations of sources. In addition, we also show the simulated amplitude spectra under excitations of monopole sound sources, for which the amplitude values in the edge sites are nearly zero in the whole spectrum, indicating that the edge states can hardly be excited by the monopole sources.

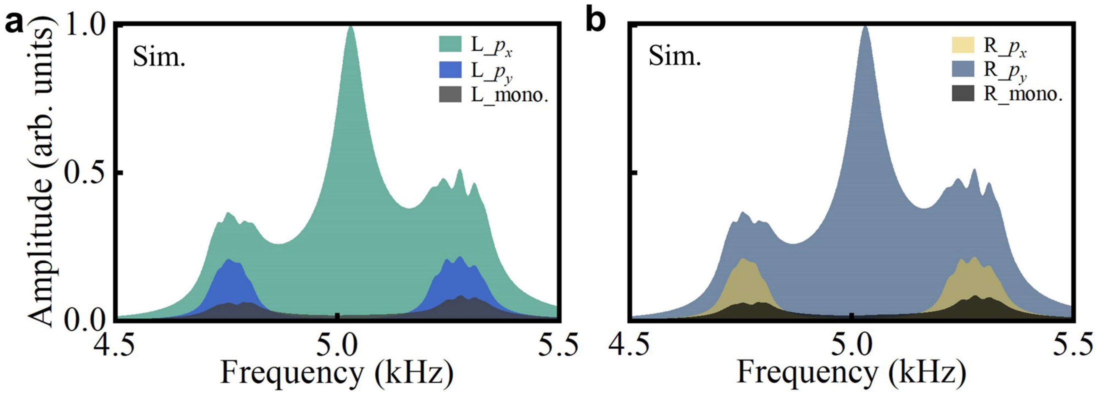

Fig. S10. (a) Simulated amplitude response spectra in the left edge site, when the orbital sound signals are generated via the L_p_x, L_p_y, and L_mono. excitations in the third site from left. (b) Simulated amplitude response spectra in the right edge site, when the orbital sound signals are generated via the R_p_x, R_p_y, and R_mono. excitations in the third site from the right.

Supplementary Note 7. *Robustness against disorders.*

In Fig. 4**d** of the main text, we have confirmed the robustness of topological orbital edge states against structural disorder by measuring the acoustic response spectra of left-side and right-side edge states excited by orbital sound sources in an aperiodic zigzag chain. For comparison with the experimental results, the numerically simulated acoustic response spectra are presented in Figs. S11**a** and S11**b**, where there exist clear prominent resonance peaks only for L_p_x and R_p_y excitations.

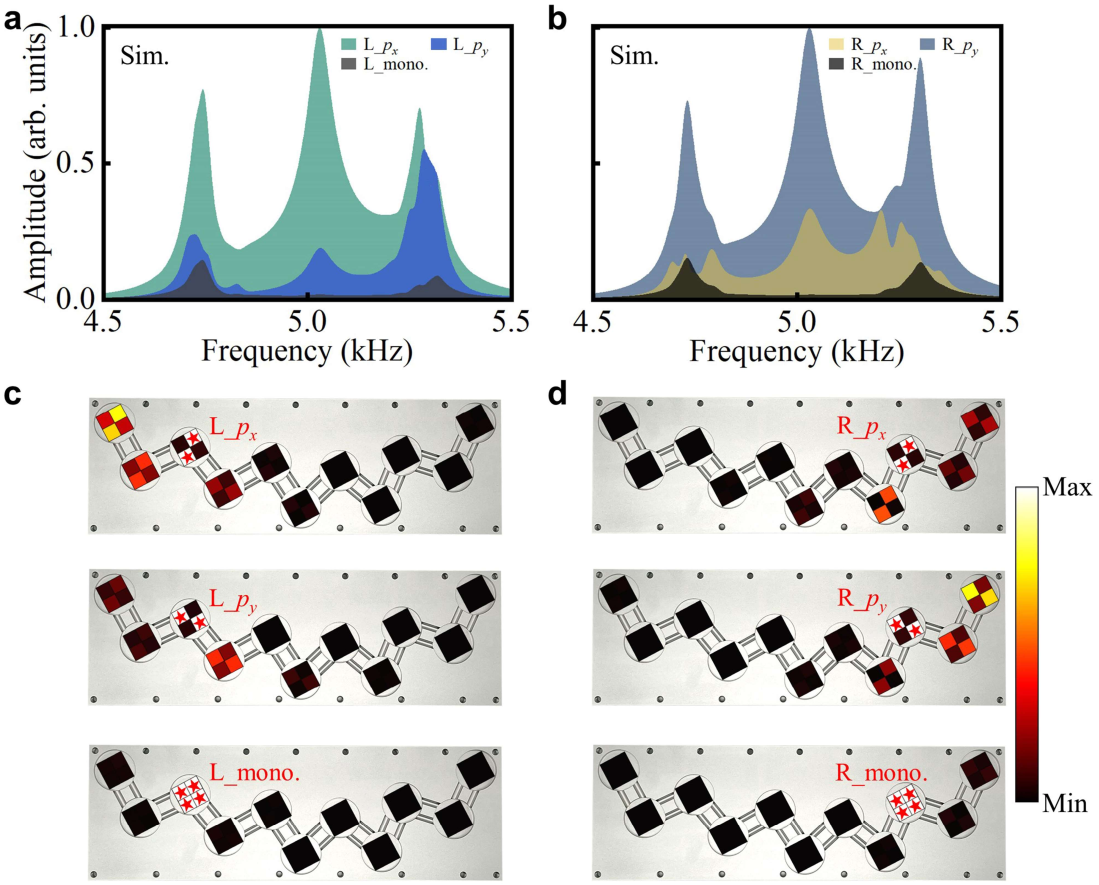

Fig. S11. (a) Simulated amplitude response spectra in the left edge site, when the orbital sound signals are generated via the L_p_x, L_p_y, and L_mono. excitations in the third site from left. (b) Simulated amplitude response spectra in the right edge site, when the orbital sound signals are generated via the R_p_x, R_p_y, and R_mono. excitations in the third site from right. (c), (d) Measured pressure amplitude distributions under the above six different excitations respectively in the disordered lattice sample. Source data are provided as a Source Data file.

In Figs. S11**c** and S11**d**, we show the measured pressure amplitude distributions under the L_p_x, L_p_y, and L_mono. excitations and the R_p_x, R_p_y, and R_mono. excitations, respectively. The red stars mark the positions where the speakers were inserted in our experiments. The results show that the orbital edge states will emerge in the edge site cavities for L_p_x and R_p_y excitations, reflected by the top-row sub-figure in Fig. S11**c** and middle-row sub-figure in Fig. S11**d**. However, for L_p_y and R_p_x excitations, the orbital edge states cannot be well excited, reflected by middle-row sub-figure in Fig. S11**c** and top-row sub-figure in Fig. S11**d**. For the monopole-type sources, orbital edge states can hardly be excited, as reflected by the bottom-row sub-figures.

To further illustrate the robustness, the on-site resonant frequency perturbations are taken into consideration, which could be the major origins of disorders in experiments because of the fabrication errors. Here, two scenarios are considered: one is that disorders are on all the sites except the left and right edge site (Scenario 1), and the other one is that disorders are only introduced on the two edge sites (Scenario 2). We perform simulations for these two scenarios. Small hard cylinders whose heights are h_c= 2 mm and radii r_c are randomly distributed in the range [0.5mm, 2.5mm], are placed at the bottom of the resonators[8] (shown in Fig. S12**a**). We separately simulate energy spectra of the zigzag chain by 50 random cases for both Scenario 1 and Scenario 2, which are shown in Figs. S12**b** and S12**c**. For Scenario 1, the eigenfrequencies of edge states clearly keep invariant in the bandgap (Fig. S12**b**), while for scenario 2, the edge states' eigenfrequencies are fluctuated (Figs. S12**c**). We furtherly display the spectra of specific lattice configurations as marked by the blue arrows in Figs. S12**b** and S12**c**, as shown in Figs. S12**d** and S12**e**. The eigenfrequencies of edge states obviously remain stable and degenerate in Scenario 1. However, in Scenario 2, the edge states still exist but the eigenfrequencies are no longer degenerate. Figure. S12**f** shows the simulated response spectra at one edge resonator in different scenarios. For Scenario 1 (bulk disorder), the resonance peak remains unchanged, while for Scenario 2 (edge disorder), the peak still exists but shifts to a lower frequency.

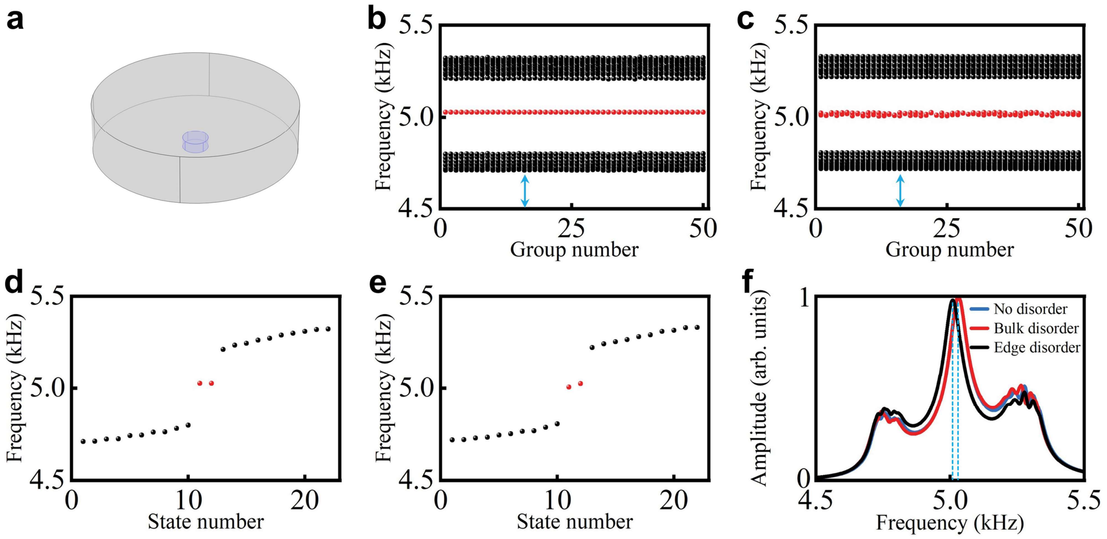

Fig. S12. (a) Schematic of the disorder introduced in the resonator. A small rigid cylinder (blue) is put at the bottom of the resonator. (b), (c) Energy spectra for 50 different lattices with rigid cylinders of random radii in the resonators, introducing the bulk disorder and edge disorder, respectively. (d), (e) The eigenfrequency spectra for specific lattice configurations marked by blue arrows in (b), (c) respectively. (f) Simulated response spectra at one edge for the chain with no disorder (dark blue line), bulk disorder (red line) and edge disorder (black line).

Supplementary Note 8. *Numerical results for the counterintuitive edge modes.*

In Fig. 5 of the main text, we have illustrated topological transitions and the emergence of counterintuitive edge modes in dimerized orbital SSH models. To clarify this feature more distinctively, we respectively measure amplitude spectra in the two edge sites as the sound sources are generated by the L_p_x, L_p_y, R_p_x, and R_p_y excitations, and the corresponding data are shown in Figs. S13**a** and S13**b**. Clearly, the acoustic amplitude spectra possess prominent peaks in the band gap, corresponding to the frequencies at which the counterintuitive left-side edge state and the predicted right-side edge state occur when sound signals are generated via L_p_x and R_p_y excitations. For L_p_y and R_p_x excitations, the corresponding spectra have no peaks in the band gap due to the orbital-dependent nature. Figure. S13**c** exhibits the measured pressure amplitude distributions via the R_p_x and R_p_y excitations as the orbital sound sources were inserted in the positions denoted by red stars. As expected, the sound waves spread into the bulk under R_p_x excitation as displayed in the left sub-figure of Fig. S13**c** while the p_y-like edge state is directly evidenced by R_p_y excitation shown in the right sub-figure of Fig. S13**c**.

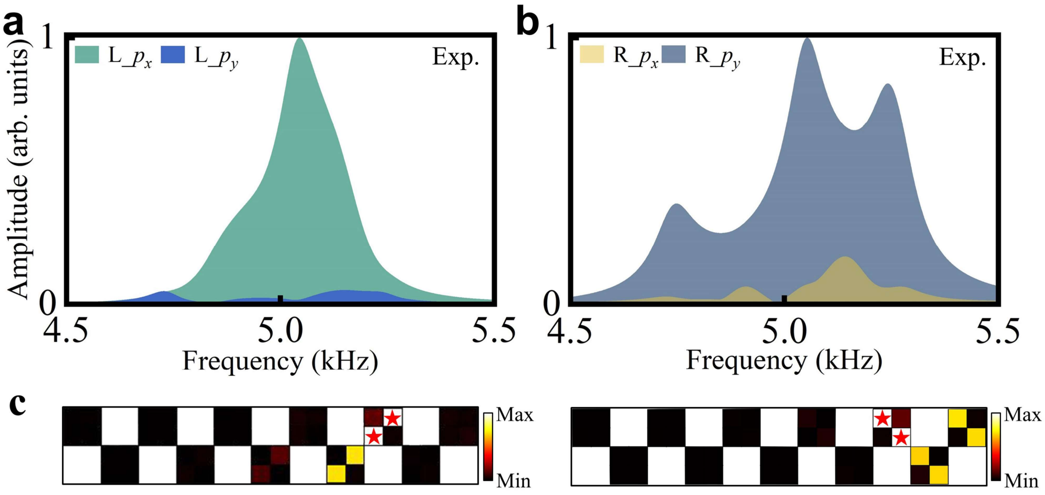

Fig. S13. (a) Measured amplitude response spectra in the left edge site as the orbital sound signals are generated via the L_p_x and L_p_y. excitations. (b) Measured amplitude response spectra in the right edge site as the orbital sound signals are generated through the R_p_x and R_p_y. excitations. (c) Captured pressure amplitude fields for the R_p_y and R_p_x excitations in experiments. Source data are provided as a Source Data file.

In comparison, the numerically simulated acoustic response spectra are obtained and displayed in Figs. S14**a** and S14**b**, where there exist evident prominent resonance peaks in the band gap only for L_p_x and R_p_y excitations, which agree with the experiments well. Moreover, we simulated acoustic pressure fields for the lattices with dimerized hopping amplitudes in Fig. 5**a** as d_2 = 0.1cm and 0.4cm respectively, which are shown in Figs. S14**c** and S14**d**. Here, degenerate p_x-like and p_y-like edge modes both emerge on the rightmost site as d_2 is 0.1cm, while the p_x-like edge mode exhibits tunneling through the bulk chain from the right to the left boundary as d_2 varies from 0.1cm to 0.4cm. Figure. S14**e** shows the acoustic pressure fields for the conventional SSH chain as the bonding angle is 180°and d_2 = 0.4cm where p_x-like edge mode retains at right termination indicating the topological transition vanish.

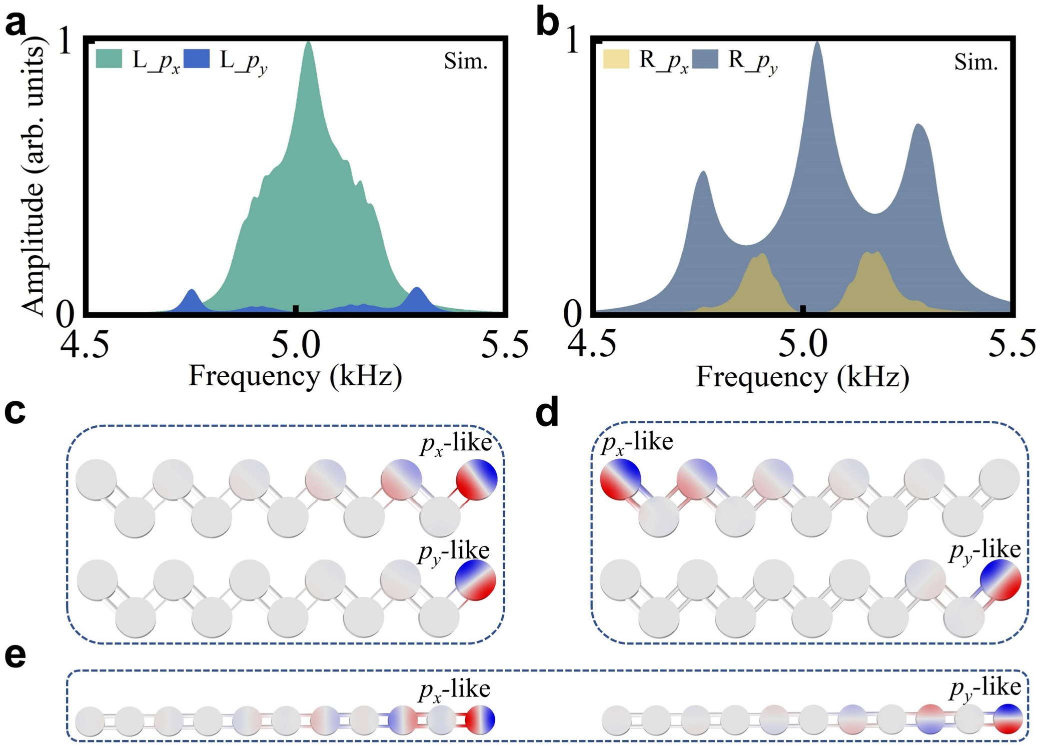

Fig. S14. (a) Simulated amplitude response spectra in the left edge site, when the orbital sound signals are generated via the L_p_x and L_p_y excitations. (b) Simulated amplitude response spectra in the right edge site, when the orbital sound signals are generated via the R_p_x and R_p_y excitations. (c) (d) Simulated eigen-fields for the lattices in Fig. 5**a** with d_2 = 0.1cm and d_2 = 0.4cm as the bonding angles keep 90°. (e) Simulated eigen-field for the conventional SSH chain as the bonding angle is 180°and d_2 = 0.4cm

Supplementary Note 9. *Details of numerical simulations and experiments.*

S9.1 *Details of the numerical simulations*

The full-wave numerical simulations are carried out by utilizing a finite element solver. In the simulation, the acoustic resonators and linked waveguides are regarded as being acoustically rigid, owing to the giant acoustic impedance mismatch between the metal Aluminum and air. Here the mass density and speed of sound in air are separately ρ_{air}=1.29 kg/m^3 and c_{air}=343 m/s . Considering the inevitable damping losses in narrow coupling tubes, we impose c'=1.0 m/s on the imaginary part to simulate the amplitude responses. The boundary condition is set to be the Bloch boundary condition for the periodic orbital orientation.

S9.2 *Details for the experiments*

All the experiments are implemented within the audible frequency range in air environment. The designed samples are manufactured by means of metal machining technique and 3D printing (the fabrication precision was ~100 μm). To facilitate the sound excitation and detection, four holes with the radius of ~2.5 mm are perforated on the top of each disk cavity, and the holes are perfectly sealed with screws when not in use. For the excitation of orbital sound in our experiment, a pair of out-of-phase sound signals are launched inside the excitation acoustic cavity, as shown in Figs. 3 and 4 of the main text. A 1/8 inch microphone (Brüel & Kjær 4138-A-15) is employed for detecting the amplitude and phase of sound waves in each disk cavity, accompanied with the other one in the same cavity as phase reference. The sound signals, recorded and processed via a network analyzer (Brüel & Kjær 3160-A-042), are utilized to map out the pressure amplitude spectra as well as the amplitude field profiles in the lattices.

Supplementary References

1. Lu, X., Chen, Y. & Chen, H. Orbital corner states on breathing kagome lattices. *Phys. Rev. B* **101**, 195143 (2020).
2. Slobozhanyuk, A. P., Poddubny, A. N., Miroshnichenko, A. E., Belov, P. A. & Kivshar, Y. S. Subwavelength topological edge States in optically resonant dielectric structures. *Phys. Rev. Lett.* **114**, 123901 (2015).
3. St-Jean, P. et al. Lasing in topological edge states of a one-dimensional lattice. *Nat. Photon.* **11**, 651-656 (2017).
4. Chiu, C.-K., Teo, J. C. Y., Schnyder, A. P. & Ryu, S. Classification of topological quantum matter with symmetries. *Rev. Mod. Phys.* **88**, 035005 (2016).
5. Delplace, P., Ullmo, D. & Montambaux, G. Zak phase and the existence of edge states in graphene. *Phys. Rev. B* **84**, 195452 (2011).
6. Fruchart, M., Zhou, Y. & Vitelli, V. Dualities and non-Abelian mechanics. *Nature* **577**, 636-640 (2020).
7. Fruchart, M., Yao, C. & Vitelli, V. Systematic generation of Hamiltonian families with dualities. Preprint at https://arxiv.org/abs/2108.11138 (2021).
8. Xue, H., Yang, Y., Gao, F., Chong, Y. & Zhang, B. Acoustic higher-order topological insulator on a kagome lattice. *Nat. Mater.* **18**, 108-112 (2019).
